# “*I Must Try Harder*”: Design Implications for Mobile Apps and Wearables Contributing to Self-Efficacy of Patients With Chronic Conditions

**DOI:** 10.3389/fpsyg.2019.02388

**Published:** 2019-10-23

**Authors:** Sharon Wulfovich, Maddalena Fiordelli, Homero Rivas, Waldo Concepcion, Katarzyna Wac

**Affiliations:** ^1^UC San Diego School of Medicine, La Jolla, CA, United States; ^2^Faculty of Communication Sciences, Institute of Communication and Health, Università della Svizzera Italiana, Lugano, Switzerland; ^3^Department of Surgery, Mohammed Bin Rashid University of Medicine and Health Sciences, Dubai, United Arab Emirates; ^4^Department of Surgery – Multi-Organ Transplantation, Stanford University, Stanford, CA, United States; ^5^Department of Computer Science, University of Copenhagen, Copenhagen, Denmark; ^6^Department of Computer Science, Université de Genève, Geneva, Switzerland

**Keywords:** self-efficacy, digital health, mhealth, self-management, technology acceptance, behavior assessment, health apps, user experience

## Abstract

**Background:**

Diverse wellness-promoting mobile health technologies, including mobile apps and wearable trackers, became increasingly popular due to their ability to support patients’ self-management of health conditions. However, the patient’s acceptance and use depend on the perceived experience and the app appropriateness to the patient’s context and needs. We have some understating of the experience and factors influencing the use of these technologies in the general public, but we have a limited understanding of these issues in patients.

**Objective:**

By presenting results from an explorative study, this paper aims to identify implications for the design of mobile apps and wearables to effectively support patients’ efforts in self-management of health with a special emphasis on support for self-efficacy of activities contributing to health.

**Methods:**

An explorative mixed-method study involving 200 chronically ill patients of Stanford Medical Center (Stanford, CA, United States) was conducted between mid-2016 and end of 2018. Amongst these, 20 patients were involved in a 4-weeks study, in which we collected the underlying wearable device use logs (e.g., Fitbit) and subjective use experience [via an Ecological Momentary Assessment (EMA)], as well as patients’ momentary perception of general self-efficacy in their natural environments and different daily contexts.

**Results:**

The results indicate that mobile apps for health and wearables have the potential to enable better self-management and improve patients’ wellbeing but must be further refined to address different human aspects of their use. Specifically, the apps/wearables should be easier to use, more personalized and context-aware for the patient’s overall routine and lifestyle choices, as well as with respect to the momentary patient state (e.g., location, type of people around) and health(care) needs. Additionally, apps and devices should be more battery efficient and accurate; providing timely, non-judgmental feedback and personalized advice to the patients anywhere-anytime-anyhow. These results are mapped on major sources of the individuals’ self-efficacy.

**Conclusion:**

Our results show how the apps/wearables that are aimed at supporting the patients’ self-management should be designed to leverage and further improve the patients’ general self-efficacy and self-efficacy of activities contributing to chronic disease management.

## Introduction

Smartphone and wearable usage is growing rapidly. Currently, more than 2.5 billion people use smartphones with this number projected to reach 5 billion in 2025 (GSM Association, 2018). Along the same trend we observe almost an exponential increase in the number of individuals diagnosed with chronic diseases like diabetes, cardiovascular disease or chronic obstructive pulmonary disease (Antó et al., 2001; Zheng et al., 2017; Piché et al., 2018). The proposed Chronic Care Model aims at better management of these individuals and includes recommendations for all different components of care, including the implementation of the health system itself, the design and delivery of decision support systems, the design of clinical information systems, the support for patient self-management, community resources and their delivery system design (Wagner et al., 2001). Smartphone and wearable devices have the potential to influence all aspects of this model, especially patient self-management support and community resources. Specifically, smartphones and wearable devices enable more convenient delivery of services for patients’ education, coaching, self-monitoring, personal goal-setting and social support.

Self-management of health implies management of activities contributing to health including physical activity, nutrition, stress, sleep, medication intake, and management of symptoms (Morgan et al., 2017; Powers et al., 2017). Self-management for chronically ill patients is highly suggested and even required, especially for patients who see their specialist occasionally and need to manage their condition daily (Wagner et al., 2001). Self-management has been shown to play a role in the reduction of disease exacerbations in chronically sick patients and improve adherence to rehabilitation (Bodenheimer, 2002; Gallagher et al., 2008; Duscha et al., 2018). This is achieved by continuous and unobtrusive monitoring of the patient’s health state while enabling the patient to follow an almost-normal daily life routine.

Self-efficacy is the self-belief that an individual can manage his/her daily life and put in effort to get the desired behavioral and health outcomes (Bandura, 1977). General self-efficacy entails general attitudes and self-beliefs to cope with a variety of difficult demands in life. Self-efficacy of health is a complex psychological concept that varies over time for the same person, according to their current health state and treatment plan. It is highly correlated with the patient’s self-management efforts and hence their health outcomes (Strecher et al., 1986; Sarkar et al., 2006; Ross and Mirowsky, 2010; Bethancourt et al., 2014). There is a large role of self-efficacy of activities contributing to the long term health state of patients (Lenferink et al., 2017; Cameron et al., 2018).

Self-efficacy has been shown to improve self-management, in direct and indirect ways. A cohort study focused on a chronic disease self-management program found that after 7 weeks of a self-management program with an emphasis on self-efficacy (including problem solving, decision making and confidence building skills), participants made statistically significant improvements in their health status, self-efficacy and health behaviors and had fewer emergency department visits (Lorig et al., 2001). This study illustrates that programs that have a self-efficacy focus can help patients improve their self-management. Self-efficacy can also improve self-management in a more indirect manner. Self-efficacy can lead to an in increase in self-belief that can spillover across other domains of life, contributing to health indirectly, e.g., having better communication and better quality of social interactions (Lauren et al., 2016). It has been shown that patients with greater quality of social interactions are more successful at self-management (Reeves et al., 2014). Additionally self-efficacy can allow individuals to build on successes, tackling easier behaviors successful and spilling over in attempting more challenging behaviors (Lauren et al., 2016). Although self-efficacy has been shown to improve self-management, the rudimentary patient education provided by the current health system is not sufficient to leverage and improve this self-efficacy, hence so many patients relapse over time (Lorig et al., 2001).

The wide use of smartphones and wearables may provide a method for increasing self-efficacy and self-management in patients with chronic conditions, although there is limited research on its efficacy (McCabe et al., 2017). However, we still know little on the role of this technology on patients’ self-efficacy of health-related activities and on if/how chronically ill patients use and experience the widely available smartphones/wearables (Wang et al., 2014; Hamine et al., 2015; McCabe et al., 2017). It has even been claimed that current so-called “gadgets” are ignoring large groups of people including physically impaired elderly and chronically ill individuals, the ones who may need the most support (Herz, 2014).

This study presents results from an explorative mixed-method study of 200 chronically ill patients at the Stanford Medical Center (Stanford, CA, United States). Amongst these, 20 patients were involved in a 4-weeks study, in which we collected the underlying wearable device use logs and subjective experience of use, as well as patients’ momentary perception of general self-efficacy in their natural environments and different daily contexts. This study is different from previous research in that it correlates *in situ* experiences with technology captured and self-reported self-management efforts, as well as and momentary self-efficacy assessments.

In Ickin et al. (2012), the authors presented factors influencing quality of experience of mobile applications in general public. The study presented in here is largely inspired by this past study (Ickin et al., 2012) and draws comparisons between its findings, but expands toward wearables in users with chronic conditions. The overlapping analyzed factors influencing the user’s experience with the mobile applications include: “application interface’s design,” “application performance,” “battery,” “phone features,” “apps and data connectivity cost,” “user’s routine,” and “user’s lifestyle”(Ickin et al., 2012). The study presented in here is bringing these results forward by showing if/how chronically ill patients use and experience the widely available smartphones apps and wearables, and what is the role of technologies in patients’ self-management of health, and specifically their self-efficacy of health-related activities.

## Methodological Approach

### Methods

Our research was purely explorative and we use a mixed-methods approach incorporating both qualitative and quantitative methods, and involving patients at different stages, via the following: (i) entry survey/open-ended interview (ii) The Ecological Momentary Assessment (EMA), (iii) The Day Reconstruction Method (DRM), and (iv) The mQoL-Log. (i) The entry survey/open-ended interview on the Stanford-based Website (RedCap), was used to collect the patients’ health state and socio-demographics, current self-management behaviors, general self-efficacy and health status, and overall attitudes toward and experiences with mobile apps/wearable (if any) for self-management (Supplementary Material). (ii) The Ecological Momentary Assessment (EMA) also referred to as Experience Sampling Method (ESM) (Hektner et al., 2007) was used to gather general self-efficacy (SE) perception (Lorig et al., 2009). It was gathered daily along one randomly selected question from 10 questions (Appendix 1) like “*Today, I can manage to solve difficult problems if I try hard enough*”(answer choices ranging from 1 “Not at all true” to 4 “Exactly true”) and was asked on smartphone screen at a random time in predefined waking hours of the morning. We designed the EMA questions such that each SE question was asked to each participant in a uniform distribution, i.e., three times along the whole study duration. Additionally, we deployed EMA to evaluate the momentary user’s experience (QoE) with a wearable/mobile app, with questions along the Mean Opinion Score (MOS) (International Telecommunication Union, 2015) for ICT-services (“*How would you rate the experience for the application*,” with answers ranging from 1 “the worst” to 5 “excellent”). They were asked during the waking hours of a day randomly after closing the fitness app (e.g., Fitbit) on the phone, i.e., the user was not able to predict when, along the app usage, the QoE question will be triggered to him/her. Each rating is purely a subjective, episodic assessment of the event provided on the basis of the given perception of the user in a given context. The EMA method contributes to capturing the momentary “ground truth” SE and user’s QoE data in the studies. (iii) The Day Reconstruction Method (DRM) (Kahneman et al., 2004) is a semi-structured interview, which enabled participants to reflect on their recent past user’s experiences and attitudes, from the past 24 hours. It puts the EMAs into context and enables us to identify the factors influencing the particular user, which may not be captured automatically. The DRM method contributes to capturing a day-level “ground truth” data in the studies. (iv) The mQoL-Log is a measurements-based Android OS smartphone logger that has been developed and validated in our lab (Manea and Wac, 2018) enabling automatic, continuous and unobtrusive gathering of the smartphone usage data (on, off, apps used, charging, WiFi/Cell ID connectivity), and details about the user’s context (physical activity e.g., walking while using phone, location derived from WiFi/Cell ID).

The interviews, surveys and DRMs data were analyzed by grouping words into clusters via an affinity clustering method. The coding and grouping of words into clusters was done by two independent coders. Inter-rater agreement was derived via a joint probability of agreement, i.e., the percentage of the time the raters agree in coding. We then derived statistics of the user’s interaction with the mobile app/wearable based on the mQoL-log datasets.

### Study Participants

We recruited 200 participants from Stanford Medical Center (Stanford, CA, United States), within the Bariatric and Metabolic Interdisciplinary Clinic or the Multi-Organ Transplantation Clinic. The Institutional Review Board of Stanford University has approved the study (IRB #29414 and #28265). All participants were informed about the goals of the study, the procedure, the data collected, stored and processed for the purpose of research, the risks and benefits of participation, and that they can withdraw from the studies and request his/her data deletion at any point in time, without any negative consequences. Each participant signed the consent form providing the above information in detail. At recruitment, all participants were assigned an anonymous identification code, which was used throughout the study. The collection of personal information (e.g., name, email addresses) was kept to a minimum. Participants were included in the study if they were cognitively intact (assessed through their understanding of the study and consent form) English-speaking adults who used Android OS smartphones daily. Many study participants (*n* = 180) only completed the open-ended interview at the beginning of the study, where they went into length about why/why not they use mobile apps/wearables for self-management. Amongst the 200 participants, the first *n* = 20 participants who had a wearable (e.g., FitBit) or a mobile application that the used for their own health self-management, committed to the 4 weeks or more (*M* = 35 ± 5 days) data collection part of the study where they answered the EMAs and completed the DRMs and had the mQoL-Log installed on their phones. The convenient sample of *n* = 20 participants was recruited by the team as a follow up and along the 4 weeks of data collection and interviews. Participants’ characteristics are shown in Table 1. None of the participants had accessibility problems related to their phone use and, when asked, none of them admitted that they are adversely affected by the beliefs regarding Electromagnetic Fields (EMF) health issues for mobile phone usage.

**TABLE 1:** Characteristics of the 20 participants collecting data for at least 4 weeks.

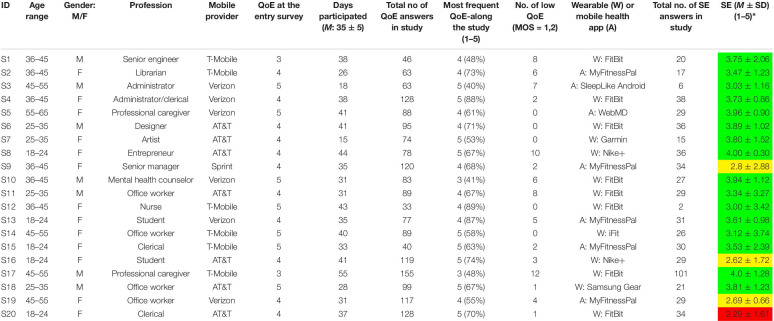

*^∗^SE is considered “very high” if its above 3 (green), “high” for 2.5–3 (yellow), “low” for 2–2.5 (orange), and “very low” for below 2 (red).*

## Results

In the following sections we present the results acquired along our study for all participants (*n* = 200), ranging from non-user or limited use and factors influencing that (section “Smartphone, Mobile Apps, and Wearables Non-Use”), to factors influencing the user’s experience (section “Use and Experience of Mobile Apps and Wearables by Patients”), to a specific focus on self-efficacy construct and our analysis how does it influence the use of technologies by patients (section “Self-Efficacy”).

### Smartphone, Mobile Apps, and Wearable Non-use

Some participants (*n* = 10) do not use technologies for self-management of health and admit it, ranging from asking for advice on how it can be used (e.g., they never heard of Fitbit) to denial of its use for their own health. An older male participant, visibly angry to be asked that question, pointed out “I don’t mix my smartphone with my health,” another female patient said “I do not want a phone reminds me about my disease” and yet another one “I have got a [Fitbit as a] gift and I dropped it.” Further, another one visibly amused by question said that “It’s all in here” [indicating his/her own head].

Some patients (*n* = 18) may have tried or have some experience with technologies contributing to own self-management, but did not use it regularly at the time our study was conducted and did not have any plans to use it in the near future. The input for these participants we got is that “Privacy is an issue,” “It’s complicated,” “I do not know how to use it,” and “I am not a techie.” These types of statements apply equally to older male and female patients.

### Use and Experience of Mobile Apps and Wearables by Patients

#### The Mobile Apps and Wearables Being Used

**FIGURE 1:**
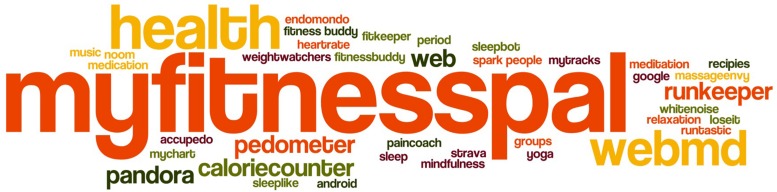
Mobile health applications used by study participants.

The rest of the participants (*n* = 172) were actively using smartphone apps or wearables for their own use. Most frequently used mobile applications were dieting applications like MyFitnessPal or calorieCounter, or exercise ones (RunKeeper, Endomondo) or the built-in Apple “Health.” As the study took place at Stanford, many of the participants (*n* = 48) mentioned Stanford Health Care “My Health” App, enabling them access to their own Stanford Health records. Additionally, they mentioned the importance of Google Search, WebMD, Facebook (for keeping in touch with loved ones), games, notes (to register symptoms), medication reminder apps, calendar, or a bible app (for mental health). Figure 1 visualizes in a word-cloud the names of health applications mentioned or used by study participants. The word-cloud visualizes the app frequency as a weighted list, as the font sizes are set in relation to the frequency of the corresponding app name.

**FIGURE 2:**
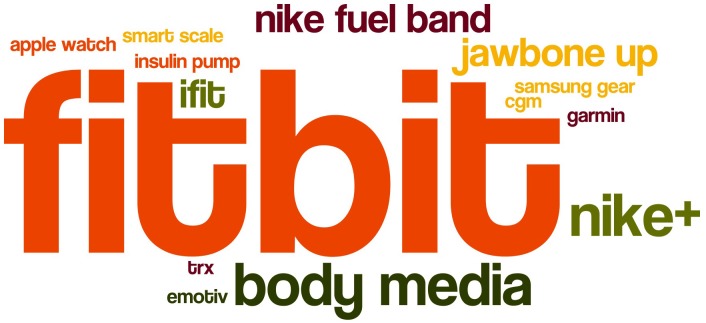
Wearables used by study participants.

Many participants (*n* = 27) mentioned that they use the built-in activity tracker apps (Apple Health and Google Fit) and treat their smartphone as a “wearable,” even if they are aware of potential inaccuracies as they sometimes forget to take along their phones. The external wearable devices were used include Fitbit, Apple Watch, iFit, JawboneUP, Garmin, Samsung Gear, Nike+. Figure 2 visualizes in a word-cloud the names of wearables mentioned or used by study participants. From the interviews we also understood that some (especially male) participants stopped using wearables like Garmin/Polar and started to use smartphone built-in activity tracker. Female participants enjoyed the playful jewelry-like design aspects of a wearable and in many cases had other band colors besides the default black for their Fitbit bands.

With respect to how much the wearables and apps were actually used, the results are as follows. From the 4 weeks study we saw that the participants who participated with their own wearable, were not frequently checking the mobile apps to see the results of their steps or calories. This was mostly because either the participants relied on notifications provided by the phone (e.g., for achieved goal of reaching predefined steps by predefined lunch time) or because these wearables have their own screen, where they can see the numbers (e.g., steps) without going into the phone app. In fact, based on datasets collected via mQoL-log we derived that the participants were interacting with a wearable (and specifically with a mobile app associated with a wearable) or a mobile app (like MyFitnessPal) on average less than 5 min a day. When asked for the context of the wearable/apps usage along the DRM, they declared that they usually use these apps when alone or as a distraction when surrounded by others (co-workers, strangers), who are not paying attention to the participant checking his/her phone. None of the participants declared their wearable/app usage and content (e.g., steps in a given day) to be a topic of the social interaction.

#### Factors Influencing Experience of Mobile Apps and Wearables by Patients

**FIGURE 3:**
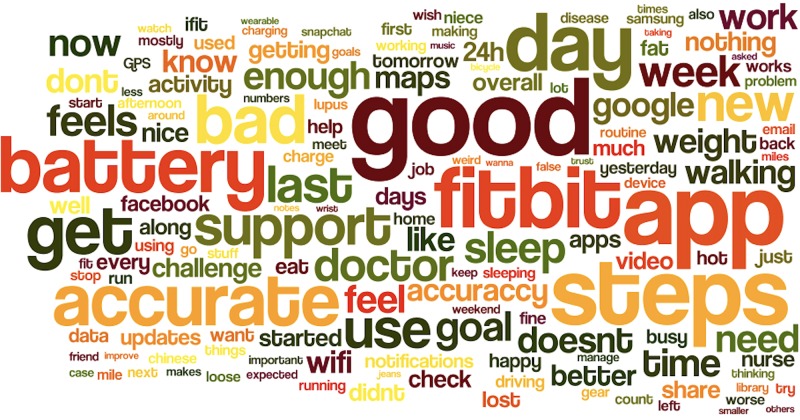
Expressions in user’s interviews and surveys.

Overall, the experience of using smartphone, apps and wearables was reported as a positive one, as even one of the patients admitted – smartphone apps “Keep me sane” (S5). Along this line, there are multitude of factors influencing the nuances of the user’s experience with mobile apps and wearables. We have used 100+ expressions from our 20 patient’s weekly DRM interviews, additional 180 patients’ interviews in the clinic. Figure 3 represents all the expressions used by study participants. We have grouped these words into clusters by using affinity clustering method, which we then labeled along the identified factor. The coding and grouping of words into clusters have been done by two independent coders, and their measure of agreement was 87%. The most disagreements were related to person’s routines and lifestyle choices, because sometimes it was about framing of e.g., notifications (interface design) or charging patterns (the battery aspect) fitting into the lifestyle choices and user routines.

We have distinguished the following factors influencing the user’s experience and discuss them in light of previous research on factors influencing the experience of commonly used mobile applications as identified in a 2012 study (Ickin et al., 2012). We first discuss the seven factors from the previous research by comparing them with previous results and expanding them toward wearables, and then add three new factors identified explicitly by this research.

##### Application and wearable interface’s design and notifications

In Ickin et al. (2012), it was pointed out that users struggled with the key positioning, web-page scrolling, and screen resizing. Additionally, users found web versions of the applications at times easier to use than mobile versions of the applications (Ickin et al., 2012).

The issue of preferring an input of long text or manual data on laptop (larger screen) is still present. Overall, the application interface in terms of application/wearable interaction was mentioned very often, yet not in negative terms of bad interface design, but in positive terms of application/wearables notifications keeping the user informed and content. Notifications provided by applications where discussed much more often than the application interface itself. Wearables’ notifications are there to fit in the routine and unobtrusively support the user’s goal matching along a day. For non-routine days, e.g., with less physical activity – the lack of notifications from wearable will be the first indicator of “bad” day in terms of matching the user’s goal: “If I do not get notifications along my afternoon, I know I am in trouble” (S12). Additionally, notifications had to be found to be informative. Notifications that did not serve any purpose resulted in participants abandoning the device (e.g., Fitbit) or turning off the notifications completely from these specific applications. An example of a helpful notification was, e.g., “Each 1000 steps I get reminded to drink water” (S10).

##### Application and wearable performance

In Ickin et al. (2012), it was pointed out that applications did not always meet user’s expectations due to typing difficulties, application settings, and network connectivity issues.

In our study, some individuals complained about applications not providing expected services or about the poor performance of their underlying network that influenced the provision of these services. A participant said, the “Internet can be touchy” (S7), to express their concern with the underlying network connectivity. Additionally, the participants complained about the lack or erroneous apps sync, low accuracy of Chinese input recognition, or inaccurate GPS. The lack of constant reliability can be a source of irritation: “When and app doesn’t work as it should, my blood pressure skyrockets with the frustration” (S5). Ultimately, participants expressed that if an application is constantly malfunctioning or not refreshing content, they will stop using the application all together.

##### Smartphone and wearable battery

In Ickin et al. (2012), it was pointed out that battery limited usage, especially later in the day.

Battery efficiency is consistently influencing the experience of the mobile users on a growing scale. Users are using more and more applications and services while continuing to expect that their phone will support them throughout the whole day. One participant was travelling and used an additional battery to support their needs. Overall, users recognized that the battery capacity issue related to usage, e.g., navigation, with screen on, GPS and continuous data and Bluetooth devices’ connectivity. Similarly, users also expected wearable charge to last at least the whole day, “if it didn’t last the full day (8 hours), then I walked for free” (S3). Additionally, a participant even mentioned that the battery time influenced her activity, “I don’t walk when [my wearable] is charging” (S2). Overall, participants mentioned having a significantly worse experience if their device did not support their full day of use.

##### Smartphone and wearable features

In Ickin et al. (2012), it was pointed out that users lacked features deemed necessary including flash player, personalized alarm clock, GPS, and privacy settings.

Mobile users noted missing features of their specific phone, which then hindered their experience, e.g., lack of or a faulty synchronization between Bluetooth devices, and lack of GPS for some older phones, lack of features for privacy settings. Additionally, participants expressed their desire for additionally features including devices that allow for greater personalization of behaviors, automatic nutrition calculations, data fusion between multiple sources (physical activity, nutrition, sleep, medications), a wearable that is waterproof, a wearable that has an ability to assess the swelling of a leg and help to self-manage the CVD patient’s fluid retention. Moreover, participants mentioned that a symptoms manager application that derived automatic checklists from notes would be a more effective way of tracking symptoms than through traditional note-taking applications. Nobody mentioned explicitly EMF issues yet two were worrying of too much synchronization, which cannot be disabled (Fitbit).

##### Apps and data connectivity cost

In Ickin et al. (2012), it was pointed out that cost of applications and data prevented user usage.

Very few participants (*n* = 15) indicated cost as a factor influencing their experience, as they mentioned their inability to purchase a smartphone or mentioned their inability to buy a wearable (*n* = 5). Only one of the participants who owned a wearable mentioned price as a factor influencing their expectations and experience: “it’s expensive, it shall work” (S7). Contrastingly, the fact that build-in apps and services are cheap and accessible was emphasized. Very few of the participants followed up with a reflection that given the affordability of the apps and services, the leak of personal data is the price to pay for having the affordable service and that the personal data privacy is definitely an issue (as mentioned earlier).

##### User’s routine

In Ickin et al. (2012), it was pointed out that different applications were used depending time of day and location, implying different mobile user need for services.

The routine of the user implied specific activities being done on weekdays and weekends and specific outcome for physical activity goal achieved at given times of the day. The users’ were most critical for their experience of smartphone/mobile apps for their non-routine days, where their decisions relied on information provided by the phone e.g., up to date location, and maps. Mobile devices play a crucial role in the organization of daily activities: “smartphone is my assistant” (S5). Mobile devices reminded users to take their medications, drink water and conduct many other important daily activities (i.e., work-related emails and calls). Mobile devices also allowed users to plan their day by looking at traffic, prices of petrol, designated routes, weather and downloading podcasts ahead of time to listen on the commute. Additionally, some participants indicated that technology helps them set up their routine, while other indicated that they wish that technology could support them in creating a better routine (i.e., better sleep choices). Contrastingly, some users did not use their devices daily. For example, a participant only used their wearable for sport activities, leaving it behind the rest of the day. Another participant only used their smartphone while traveling, leaving it behind when at home or at the office.

##### User’s lifestyle and identity

In Ickin et al. (2012), it was pointed out that users used specific applications that supported their lifestyle choices (i.e., nutrition, exercise) and that were found to be convenient.

Mobile devices and wearables form an important part of users’ lives. Applications are there to support healthy (ex. physical activity) or unhealthy (ex. being “lazy” and watching videos on the phone). They can also be modified to fit a user’s routine: a clip being put in a pocket, clip (on bra) for users working (ex. in a production line) where they are not allowed to have anything on a wrist; watch for people who are active and wish to get updates on their steps throughout the day (especially female). Besides design modifications for routine, there is also esthetic modifications. Participants mentioned the need for “showing off” to others (via colorful choice of Fitbit bracelet, females mostly, e.g., S4) or hiding from others that a wearable is used (mostly male users). Lifestyle choices are also related to listening to educational/informative podcasts of audiobooks via a smartphone while commuting and having an adequate support for that. Without smartphones, many participants reported feeling “naked” or when a wearable is lost a patient expressed that she “lost her life” (S12).

##### Smartphone apps and wearable accuracy

This factor was not mentioned in the 2012 paper (Ickin et al., 2012) and constitutes new finding. The accuracy or even a perception of inaccuracy of wearables consistently influences the experience of some of its users. The fact that devices are inaccurate (e.g., Fitbit for assessment of biking) and provide erroneous data (e.g., device registers steps, while the user is driving on a bumpy road) has been noticed by many participants. On the one hand, the users are fully aware that these are not medical accurate devices and they would not expect the medical practitioner to trust only the data acquired to make a clinical decision. Some participant expressed clearly that the device is “accurate enough to recognize my efforts [in exercising more].” On the other hand, the users do not accept the numbers without a critical perspective, and they notice the errors in data. Some participants dropped the wearable use because it was too unreliable for them, e.g., when it consistently did not account for their biking, the user dropped it. One female patient pointed out that her Fitbit experience was fine for few days until she discovered its inaccuracy by comparing it to another wearable: “I have compared [Fitbit] to my husband’s Garmin and I was disappointed” [and have stopped using it after a total week of use]. For some interviewed participants who did not own any wearables, the doubt in accuracy of the device became an “excuse” for not using at all.

##### Willingness to share data with others

This factor has not been mentioned in 2012 paper (Ickin et al., 2012) and constitutes new finding. The study participants were also asked if they are sharing or not their wellness/fitness tracking data from apps and wearables with others and why (or why not). Overall, most of the patients rather not share the data, as they expressed the fact that they are not expecting to compete with others. They fear that sharing this information with friends or family could instead of making the patient feeling better – makes him/her feel worse and inadequate about their progress. One participant pointed out that she feels “enough people are judging offline” (S4); meaning that she is experiencing enough judgment about her condition (obesity) in her daily life and she prefers not be judged online as well. She uses her Fitbit by herself and for herself only.

If the user mentions that he/she shares data with others, they are usually family members, same age and potentially aiming for the same goals (e.g., nutrition or physical activity). The persons with whom the data is shared are there to support the participants’ self-management efforts; while the participant supports their efforts. An example is a participant (also a diabetes patient) who shares Fitbit data via setting up competitions with his younger niece living in other state across the country and suffering from bipolar disorder. He pointed out that he knows that physical activity is recommended in her state and he is supporting her from far away.

When commenting on wherever they would share the data with their medical team, they, on the one hand, again point to accuracy, “doctor wont’ trust the data.” On the other hand, some participants commented that they had not received negative feedback from their medical teams (nurses and clinicians), in contrary, the inputs were positive like “whatever it takes to get you more healthy” or “whatever contributing to your health is good.” The participants would be generally accepting to share the data with their doctors.

### Self-Efficacy

This factor has not been mentioned in 2012 paper (Ickin et al., 2012) and constitutes new finding, and since is related to the core aim of this paper is presented in a separate section and in great detail. Self-efficacy was a frequent theme of conversation with the participants; discussing their belief that they can manage their own physical activities, nutrition, sleep, medication adherence and other activities contributing to their health self-management with (or without) help of the mobile apps and wearables. There are four main sources of self-efficacy: (i) own past experiences, (ii) experiences of people similar to ourselves, (iii) verbal encouragement from others, and (iv) intrinsic state (Bandura, 1977, 1997). We analyzed the participant data for these factors and present the design implications for mobile apps and wearables contributing or hindering self-efficacy.

#### Past Experiences

Wearables and mobile device applications are designed to allow users to easily visualize trends. It allows users to see their previous successes as well as previous failures. Although wearables and apps enable users to see the trends, the target levels are predefined. For example, the target step levels are usually at 10,000 steps and some participants admitted that even though that set target was too high for them and they had never been able to achieve this predefined goal, they were not “tech savvy” enough to know how to change the goal. The self-defined targets do not encourage subsequent increases in effort. It was suggested by Kate Lorig verbally that wearable or apps should first monitor the user (based on passive monitoring) and then suggest a feasible goal. This more flexible goal setting, based on user goals, past experiences, trends should allow any user, regardless of their technical skills to benefit, adjust and feel good about achieving their goals.

#### People Similar to Ourselves

Social networks should theoretically allow and facilitate users to find people living a similar lifestyle and undergoing similar challenges. Although that can be true in some cases, social networks self-representation are not always accurate (Mallan, 2009). Therefore, it can be hard to find people going through similar challenges, not because people are not going through these challenges but because many people may not be posting about it. Weakness, vulnerability, real daily challenges is not the content people want others to see. Additionally, it is not content that people are willing to share due to fear: “enough people judging me offline” (S4). This can make it hard to find other individuals with similar chronic conditions, going through similar daily challenges. Social networks do not always promote self-efficacy, if you feel that everyone is doing better than you and having an easier time with the challenges or if you don’t identify with your social group. The design implication is to enable users to connect with others of the same/similar characteristics. Observing others achieving specific goals that we may identify ourselves with, may increase self-efficacy of the individual users.

#### Verbal Encouragement From Others

**FIGURE 4:**
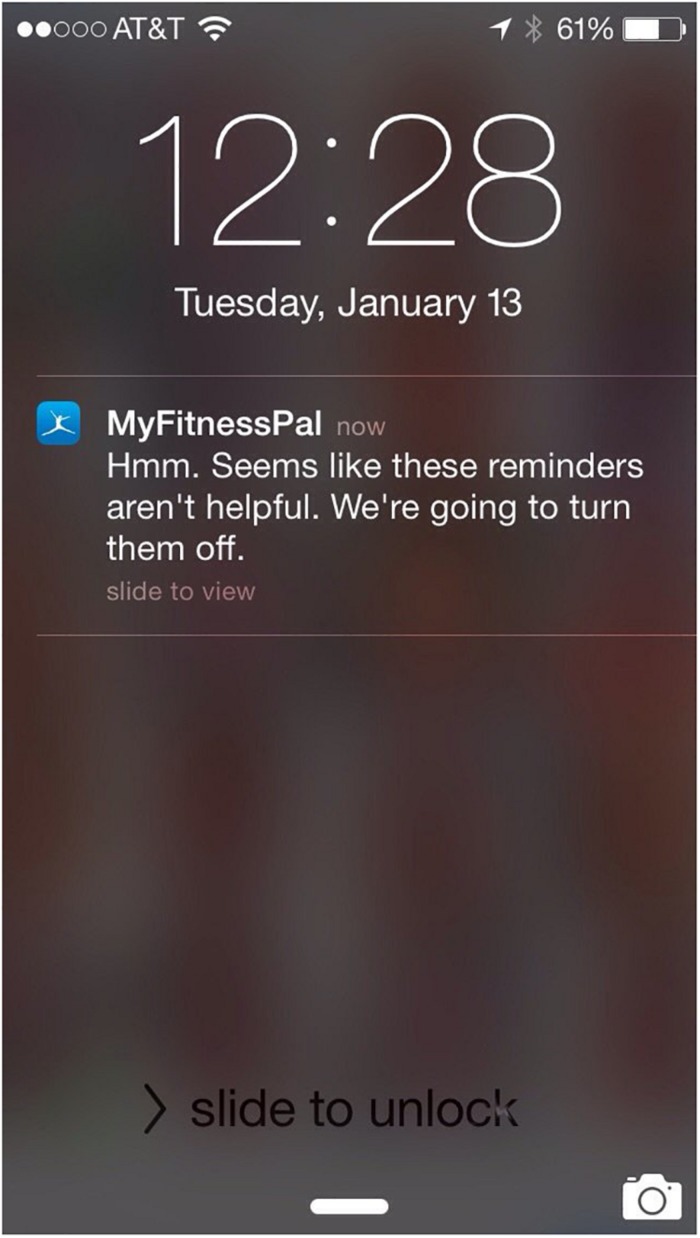
MyFitnessPal coaching feature turning off after not logging in for a few days.

There are many applications and wearables that send motivational notifications to users after achieving a certain goal or to remind the user to continue tracking (i.e., track the next meal). These notifications provide users with a reminder to and sometime encouragement to continue their hard work in achieving their goal. Although a lot of these notifications can be helpful, one participant pointed out that the MyFitnessPal coaching feature is “giving up on her” (S9) when she has not logged for few days. That was discouraging and she pointed out that it could be redesigned better – the device should not give up on an individual ever, as it really feels judgmental and discourages the person from progress (Figure 4). Now it’s kind of supporting the patients’ relapse and that’s “sad” as the patient said, “The app shall be last one to give up on the patient” (S9).

#### Individual’s Intrinsic State

Current evidence in behavioral medicine and health psychology shows that habits are hard to change (Ouellette and Wood, 1998). It can be hard to start a change and keep it going. Additionally, it can be hard to fail at a new habit and have the courage to restart from the beginning. Wearables and mobile devices allow users to start a goal again and again. It allows the user to suggest their own self-belief in efforts to be put into self-management. For example, a participant said that this feature allowed them to “Know that if I failed yesterday to meet physical activity goal, I can start again tomorrow” (S18). Another participant further echoed this, “I can do 5000 [steps] in a good day. If I do not, I can try again tomorrow.” This shows that the ability to keep trying allows people to set up the intrinsic motivation to try again tomorrow. Additionally, some participants admitted that the wearable or application was not at fault for their inability to consistently reach a goal: “I Must try harder[…] Device is not faulty of my behavior” (referring to weight gain) (S1).

Additionally, participants reported that their emotional state influenced their daily interactions with the apps/wearables. For example, a participant (S20) discussed that they received the device as a gift. Since it was a gift and something, they did not intend to purchase they do not use it as often. The importance of emotional state was also discussed in terms of daily interactions, participants expressed that they had momentary expression that a wearable is a “friend” or “enemy” depending on if they had met the goal (and felt good) or not (and felt bad).

## Limitations

Limitations of this study include the fact our sample was a convenience sample, included only self-selected participants, limited time and we did not log the content on the health app or wearable used. In terms of the convenience sample, the researchers were only available to talk to participants at certain times of the day, this may have influenced the recruited participants. Participants were self-selected to participate in this study, and this could have influenced the study pool for people with a particular positive or negative view on wearables and applications. We are not able to quantify this bias. Additionally, due to the structure of the clinic, sometimes time was more limited with specific participants. Moreover, participants perceived the EMAs for self-efficacy differently. Some perceived them as encouraging, while some thought these were annoying, as they were tired in the morning and were not in a mood for challenging questions.

## Conclusion and Areas of Future Work

Our study illustrates that applications and wearables that are aimed at supporting the users’ self-management should leverage and further improve the patients’ self-efficacy of activities contributing to self-management of chronic disease. The results indicate that mobile apps for health/wearables have potential to enable better self-management and lead improved wellbeing but must be further refined to address different human aspects of their use. Specifically, the apps/wearables should be easier to use, more personalized and context-aware for the patient’s overall routine and lifestyle choices, as well as with respect to the momentary patient state (e.g., internal feeling of self-efficacy – achieved some goals or not) and health(care) needs. Additionally, apps and devices should be more battery efficient and accurate; providing timely, non-judgmental feedback and personalized advice (matching current state and bringing the patient to nest state slowly, along personal relapses and achievements) to the patients anywhere-anytime-anyhow.

Improved belief increased health outcomes, and we must re-engineer technologies to better engage the ones who need the solution the most and even further improve this belief (via increased arousal and self-gamification) and co-design with patients (Norman and Draper, 1986; Schuler and Namioka, 1993; Sanders and Stappers, 2008) (to leverage the self-efficacy sources). The self-efficacy source (people like ourselves) is harder because it depends on others’ behavior and how people display those behaviors, this is now over-emphasized in current designs. Current designs assume people want to constantly compete, while some patients do not want or welcome that pressure. Addressing this would require adapting the designs to different personality traits (e.g., openness, agreeableness and neuroticism), which could then be translated into interaction types leading to an increase in the individual’s self-efficacy. Knowing what we know now, mobile apps and wearable devices should be designed to leverage and improve the individual’s self-efficacy as much as possible to lead to the user to the healthier behaviors in subtle design-based ways.

All our findings point toward a strategy that is well known in the health communication field, which is tailored communication (Hawkins et al., 2008). The interest in increasing self-efficacy because of its potential to positively influence self-management of chronic condition has been clearly stated. Our findings, however, show that pre-conceived design ideas aimed at influencing self-efficacy do not correspond to what users want, as we show the necessity of having a design adapted to different personality traits in order to increase individual’s self-efficacy. But also beyond self-efficacy, our research shows how user experience could be positively influenced and sustained by personalized solution in apps and wearables, as well as by personalized feedback. Many behavioral interventions have shown that one size does not fit all and in order to become able to influence behavior we need to tailor the solutions to the individual needs. Mobile and wearables offer an unprecedent opportunity to tailor communication, as personal devices are able to collect data that can be used to this end. If tailored approach has been already proven successful on the web (Lustria et al., 2013), mobiles and wearable have the moral duty to bring this a step forward. This design implication is fundamental for the future.

## Data Availability Statement

The datasets generated for this study will not be made publicly available due to study IRB and HIPAA. Requests to access the datasets should be directed to the corresponding author.

## Ethics Statement

The studies involving human participants were reviewed and approved by David D. Oakes, M.D., Stanford University (Protocol 28265) and Michael D. Amylon, M.D., Stanford University (Protocol 29414). The patients/participants provided their written informed consent to participate in this study.

## Author Contributions

KW collected the data and coded it (with MF). KW and SW wrote the first draft of the manuscript. SW, KW, HR, WC, and MF contributed to all versions of the manuscript.

## Conflict of Interest

The authors declare that the research was conducted in the absence of any commercial or financial relationships that could be construed as a potential conflict of interest.

## Appendix 1: General Self-Efficacy Scale

The participants were asked daily 1 general self-efficacy question:

1. Today, I can manage to solve difficult problems if I try hard enough.
2. Today, if someone opposes me, I can find the means and ways to get what I want.
3. Today, it is easy for me to stick to my aims and accomplish my goals.
4. Today, I am confident that I could deal efficiently with unexpected events.
5. Today, Thanks to my resourcefulness, I know how to handle unforeseen situations.
6. Today, I can solve most problems if I invest the necessary effort today.
7. Today, I can remain calm when facing difficulties because I can rely on my coping abilities.
8. Today, if I am confronted with a problem, I can find several solutions.
9. Today, if I am in trouble, I can think of a solution.
10. Today, I can handle whatever comes my way.

Response options:

1 = not at all true (low self-efficacy) 2 = hardly true 3 = moderately true 4 = exactly true (high self-efficacy).
