# Supplementary material for: “I Must Try Harder”: Design Implications for Mobile Apps and Wearables Contributing to Self-Efficacy of Patients With Chronic Conditions
Source: Front Psychol. 2019 Oct 23;10:2388. doi: 10.3389/fpsyg.2019.02388 (PMC6842939; doi:10.3389/fpsyg.2019.02388)
Supplement: Supplementary file 1 [file Data_Sheet_1.PDF]

How do you experience your smartphone?

1. Information for Study Participants

***1. This survey is part of a research study conducted by Dr. Homero Rivas and Dr. Katarzyna Wac at Stanford University.**

Purpose of this Study
As smartphone use has become a common part of our lives in various activities, the goal of this study is to gather data on smartphone usage and users experiences and expectations for, e.g., applications used, wearables used, and phone battery lifetime. By studying this, we hope to create guidelines to help designers of applications for these smartphones to provide a stress-free experience.

Procedures
You will be asked to complete an online survey to ensure that you qualify. The estimated time for the survey is 20 minutes.

Participant Requirements
Participation in this study is limited to individuals ages 21 and older, and who own and use an Android OS smartphone.

Risks and Benefits
The risks and discomfort associated with participation in this study are no greater than those ordinarily encountered in daily life or while carrying a cell phone. The benefits, which may reasonably be expected to result from this study, are understanding of your smartphone usage experience and expectations, understanding a use of a wearable device and understanding of own habits related to the use of these. We cannot and do not guarantee or promise that you will receive any benefits from this study.

Compensation & Costs
There is no compensation on completing this study and there will be no cost to you if you participate in this study.

Confidentiality
The data captured for the research does not include any personally identifiable information about you. Your IP address will not be captured.

Voluntary Participation

If you have read this form and have decided to participate in this project, please understand your participation is voluntary and you have the right to withdraw consent or discontinue participation at any time without penalty or loss of benefits to which you are otherwise entitled. You have the right to refuse to answer particular questions. Your individual privacy will be maintained in all published and written data resulting from the study. The results of this research study may be presented at scientific or professional meetings or published in scientific journals.

Right to Ask Questions & Contact Information
If you have any questions, concerns or complaints about this research, its procedures, risks and benefits, contact the Protocol Director, Homero Rivas , (650) 721-2757.

If you are not satisfied with how this study is being conducted, or if you have any concerns, complaints, or general questions about the research or your rights as a participant, please contact the Stanford Institutional Review Board (IRB) to speak to someone independent of the research team at (650)-723-5244 or toll free at 1-866-680-2906. You can also write to the Stanford IRB, Stanford University, MC 5579, Palo Alto, CA 94304.

Alternate Contact: If you cannot reach the Protocol Director, please contact Katarzyna Wac .

The Stanford University Institutional Review Board (IRB) has approved the use of human participants for this study under the protocol number IRB-29414.

- ☐ I am age 21 or older.
- ☐ I have read and understand the information above.
- ☐ I want to participate in this research and continue with the survey.

2. Your Mobile Phone Usage

*2. In which ***year*** have you started to use a mobile phone for ***the first time***? (e.g., 1998)

*3. Do you have a fixed-line phone (i.e., landline) at home?

- yes
- no

*4. What kind of mobile phone(s) do you use today? (select all that apply)

- «classic» mobile - just voice and SMS
- «smart» mobile - voice, SMS, web, email, applications, camera etc.
- none

Other (please specify)

*5. Please specify the model of (smart)phone(s) you use today (e.g., iphone, samsung s4)

6. If you have multiple mobile phones (mentioned above), which of those is your ***primary phone***?
Please specify the phone model:

7. If Android OS phone is your primary phone, which firmware version does it run? (see "Settings" -> "About device" -> "Android version", e.g., version 4.2.2, 4.5)

* 8. Which is the network provider you use for your primary phone? (select one answer)

- verizon
- at&t
- sprint
- t-mobile
- cricket
- vodafone
- orange
- movistar
- vodafone
- O2
- 3
- swisscom
- sunrise
- telenor
- TDC
- Telia
- other (please specify)

* 9. Is your primary phone...

- a personal phone?
- a professional (business-owned) phone?
- other (please specify)

3. Your Mobile Phone Usage (cont.)

***10. What is the billing method you use for your primary mobile phone?**

- ◯ monthly billing (post-paid)
- ◯ pre-paid (card)
- ◯ other (please specify)

***11. Do you have a data plan (i.e., Internet option) for your primary phone?**

- ◯ yes
- ◯ no
- ◯ not sure

12. If you have data plan (i.e., Internet option) for your primary phone, how much do you pay for it (monthly)?

***13. When your primary phone is fully charged, its typical battery life (without subsequent charging) is... (please specify in week(s) and/or day(s) and/or hour(s)).**

***14. On average, how frequently do you charge the battery of your primary phone?**

- ◯ more often - multiple times per day/night
- ◯ every day/night
- ◯ once every 2 days
- ◯ once every 3 days
- ◯ once every 4 or more days
- ◯ other (please specify)

15. ***When do you usually charge your primary phone?**

- ◯ day
- ◯ night
- ◯ other (please specify)

16. ***Where do you usually charge your primary phone?**

- ◯ home
- ◯ work/school
- ◯ car
- ◯ other (please specify)

***17. Do you feel that the lifetime battery life of your primary phone is long enough?**

- ◯ yes
- ◯ no, i wish it to be ... please specify in week(s) and/or day(s) and/or hour(s):

18. How ***many hours per a day (i.e., per 24h) you estimate your primary phone is NOT in your close proximity, i.e., it is NOT in your pocket or NOT at your arm distance?**

***19. In regards to Question 18: Do you think that the proximity of your primary phone to you differs between a weekday and a weekend?**

- ◯ no
- ◯ yes it differs (please specify how)

***20. In regards to Question 18: Do you think that the proximity of your phone to you differs between between your home and office/school locations?**

- ◯ no
- ◯ yes it differs (please specify how)

21. If you are moving ***inside your home do you usually...**

- ◯ carry your phone around (e.g., in the pocket, bag)?
- ◯ place your phone in a fixed place (please specify)

22. If you are ***outside your home, where do you usually carry your primary phone? (e.g., in the pocket, bag)**

23. Do you use any connectivity accessories for your phone (at least once a week)?

- ☐ none
- ☐ Bluetooth headset
- ☐ hands-free set in a car
- ☐ other (please specify)

***24. Do you use the silent or vibrate-only mode on your primary phone on a regular basis (i.e., at least once a week)?**

- ○ yes
- ○ no

***25. On average, how frequently do you use WiFi/WLAN on your primary phone?**

- ○ every day
- ○ once every 2 days
- ○ once every 3 days
- ○ once every 4 days
- ○ once every 5 days
- ○ once every 6 days
- ○ once every 7 days
- ○ less often
- ○ never
- ○ other (please specify)

4. Your Mobile Applications Usage

26. What applications *** do you currently use on your primary phone and with which frequency (on average)? (select all that apply)**

	everyday	once a week	once a month	less often	never or N/A
Voice telephony	○	○	○	○	○
Voicemail	○	○	○	○	○
SMS (texting)	○	○	○	○	○
MMS	○	○	○	○	○
Web browsing	○	○	○	○	○
Web: blog reading	○	○	○	○	○
Web: blog writing	○	○	○	○	○
Web: social network (e.g., twitter)	○	○	○	○	○
E-mail	○	○	○	○	○
Alarm clock / stop watch	○	○	○	○	○
Calculator	○	○	○	○	○
Maps / navigation (e.g., GPS)	○	○	○	○	○
Games	○	○	○	○	○
Ringtones	○	○	○	○	○
Music (e.g., MP3)	○	○	○	○	○
Radio	○	○	○	○	○
Photos and videos capturing (camera)	○	○	○	○	○
Photos and videos download or upload	○	○	○	○	○
Voice recording (dictaphone)	○	○	○	○	○
Calendar/Scheduling	○	○	○	○	○
ToDo list/Notes	○	○	○	○	○
Office (e.g., Word, Excel)	○	○	○	○	○
Payment via SMS	○	○	○	○	○
Chat /Instant message (e.g., WhatsApp)	○	○	○	○	○
VoIP (e.g., Skype)	○	○	○	○	○
e-banking	○	○	○	○	○
financial services (e.g., mobilpay)	○	○	○	○	○
Online shopping	○	○	○	○	○
Mobile TV	○	○	○	○	○
Other	○	○	○	○	○

(please specify)

27. How much time on average you spend on your primary phone for ***voice calls per a day (i.e., calling somebody and being called)?**

- ◯ 0 - 5 minutes
- ◯ 6 - 10 minutes
- ◯ 10 - 15 minutes
- ◯ 15 - 30 minutes
- ◯ 30 minutes - 1 hour
- ◯ 1 - 3 hours
- ◯ 3 - 5 hours
- ◯ 5 - 8 hours
- ◯ more than 8 hours
- ◯ other (please specify)

5. Your Overall Experience and Expectations

28. How would you rate your ***overall experience of use of your mobile phone?**

	very bad	bad	just 'ok'	good	very good
your experience	◯	◯	◯	◯	◯

feel free to justify your rating below

29. Which of your expectations for your mobile phone or applications have not been met (yet)? (please specify)

6. Your quality of life experience and expectations

***30. How would you rate your quality of life?**

	very poor	poor	neither poor nor good	good	very good
Your quality of life	◯	◯	◯	◯	◯

What counts for your quality of life? Feel free to express yourself and justify your rating.

31. Which of your quality of life expectations have not been met (yet)? (please specify)

32. What application(s) * do you currently use*** on your mobile phone that contribute to your quality of life? How do you use them? How do they contribute to your quality of life?**

***33. How satisfied are you with your health?**

	very dissatisfied	dissatisfied	neither satisfied nor dissatisfied	satisfied	very satisfied
Your health	◯	◯	◯	◯	◯

What counts for your health? Feel free to express yourself and justify your rating.

34. What application(s) * do you currently use*** on your mobile phone that contribute to your health? How do you use them? How do they contribute to your health?**

35. Think of an event when you had a particularly ****satisfying experience with your phone contributing to your health and/or care needs**. When did this experience occur? Describe the events leading up to this experience. Describe, as best as you can, the situation, the experience with your phone and what made it satisfying. This answer can relate to some specific experience with the application(s) mentioned in question 32/34, or it can relate to another experience with your phone.**

36. Think of an event when you had a particularly ****dissatisfying experience with your phone contributing to your health and/or care needs**. When did this experience occur? Describe the events leading up to this experience. Describe, as best as you can, the situation, the experience with your phone and what made it dissatisfying. This answer can relate to some specific experience with the application(s) mentioned in question 32/34, or it can relate to another experience with your phone.**

37. Do you use any ***external sensors/wearables* for monitoring some aspects of your wellbeing/health or behaviour (e.g., exercise, sleep, nutrition, moods)?**

38. If you have answered 'yes' above, how would you rate your ***overall experience* of use of your ***external sensors/wearables***?**

	very bad	bad	just 'ok'	good	very good
your experience	◯	◯	◯	◯	◯

feel free to justify your rating below

39. Do you share any of the data collected through your wearables/apps with your healthcare provider?
If 'yes' what was/is the reaction and appreciation from their side?
If 'not' - why not?
Feel free to tell us more.

40. If you have answered 'yes' in question 37, which of your expectations for your your ***external sensors/wearables* have not been met (yet)? (please specify)**

7. Your daily life experiences

***41. Choose the answers you identify with most. Go with your first instinct rather than thinking too hard. There are no right or wrong answers.**

	Not at all true	Hardly true	Moderately true	Exactly true
I can always manage to solve difficult problems if I try hard enough.	◯	◯	◯	◯
If someone opposes me, I can find the means and ways to get what I want.	◯	◯	◯	◯
It is easy for me to stick to my aims and accomplish my goals.	◯	◯	◯	◯
I am confident that I could deal efficiently with unexpected events.	◯	◯	◯	◯
Thanks to my resourcefulness, I know how to handle unforeseen situations.	◯	◯	◯	◯
I can solve most problems if I invest the necessary effort.	◯	◯	◯	◯
I can remain calm when facing difficulties because I can rely on my coping abilities.	◯	◯	◯	◯
When I am confronted with a problem, I can usually find several solutions.	◯	◯	◯	◯
If I am in trouble, I can usually think of a solution.	◯	◯	◯	◯
I can usually handle whatever comes my way.	◯	◯	◯	◯

Fell free to justify your answers below

8. Tell us about yourself

***42. What is your gender?**

◯ female

◯ prefer not to say

◯ male

***43. What is your age?**

◯ 18-24

◯ 25-35

◯ 36-45

◯ 45-55

◯ 55-65

◯ above 65

***44. What is your occupation?**

[ ]

***45. What is your highest level of education?**

[ ]

***46. What is your citizenship?**

[ ]

***47. What is your country of residence?**

[ ]

***48. What is your marital status?**

◯ single

◯ married or in a couple (living together)

◯ married or in a couple (living apart)

◯ widowed

◯ divorced / separated

Other (please specify)

[ ]

***49. How many people live with you?**

[ ]

***50. Thank You for your help.**

Please specify your name and email (which you can receive on your smartphone) such that we contact you directly for qualification for the "Stanford Wearables Study".

Below you can also express your questions and/or additional comments for the survey.
